# Notes on Placenta Prævia, with Report of a Fatal Case

**Published:** 1879-02

**Authors:** E. O. F. Roler

**Affiliations:** Professor of Obstetrics, Chicago Medical College


### Original Communications.

---

Article II.

### Notes on Placenta Prævia, with Report of a Fatal Case.

By E. O. F. Roler, m. d., Professor of Obstetrics, Chicago Medical College. Read before the Chicago Gynæcological Society, Dec. 27th, 1878.

The importance of placenta prævia as a dangerous complication of labor, will be my apology for selecting it as the topic for discussion this evening.

No other complication in midwifery involves greater peril to mother and child; none other demands prompter action more vigilant watchfulness and discriminating judgment. And yet the treatment is by no means definitely settled. All methods thus far adopted are liable to fail in good results, and we are yet in uncertainty as to the comparative value of the modes of treatment which have been especially recommended for the prevention of hæmorrhage.

Among authorities, opinions have varied with regard to the exact situation of the placenta on the inner surface of the uterus; and the different views entertained concerning the vascular relations which obtain between its structure and the maternal system have determined, to a considerable extent, the management of these cases during labor. The subject, however, is of vast proportions, and I do not propose attempting an elaborate or even systematic discussion. I intend simply as preliminary to the report of a fatal case which came under my care, to mention a few of the more important points embraced in the subject, making comments thereon as expressions of my own opinion, and leave the further discussion to the members of the society.

The possibility of the descent of the ovum beyond its strictly normal position in the uterine cavity, must depend on two accidental circumstances:

1st. Failure to make *prompt* attachment near the Fallopian tube after its entrance into the cavity. This might be owing either to a faulty deciduous formation at the time of its arrival, or it is possible that the ovum, not becoming fecundated until a later period, would find neither the mucous surface prepared for its reception, nor its own vitality sufficiently changed to admit of adherence.

2nd. The mucous surfaces of the body of the uterus must be separated to such an extent as to furnish an easy passage through its cavity. This condition is less likely to exist in primiparæ, and statistics prove that, as a class, they are exempt from this accident. Inflammatory conditions of the mucosa would especially favor this patent state of the cavity of the organ. These conditions being present, gravity, aided possibly by the flow of secretions, would carry it downward beyond the zone of normal attachment.

### LOCALITY OF PLACENTA PRÆVIA.

Authorities are not agreed in regard to the possibility of attachment of the ovum within the canal of the cervix.

Barnes, in his "Obstetric Operations," makes his theory and his practice contradictory. He agrees with Stoltz and Mathews Duncan in the opinion that the cervix is in no way involved in the reception of the ovum, while later, in making his imaginary zones, he makes the orificial, or cervical region, the seat of dangerous attachment for the placenta.

Rigby and Lee, among the older writers, Cazeaux and Bell among the more recent, insist that the ovum occasionally finds an intra-cervical location.

In a patent state of the os internum this would seem to be possible. The neck, however, is as separate anatomically as it is functionally from the body of the uterus. The mucous membrane of its cavity is entirely unlike the cavity of the body, and does not participate in the changes which take place in the latter in the event of fecundation of the ovum. As a consequence, the deciduous formation is entirely absent. Admitting, however, its growth in this situation, abortion at an early day must be inevitable. In the process of expansion of the neck, the os internum would as effectually prevent invasion upward as it does downward when the ovum is lodged within its normal cavity.

The os externum having less power of resistance would soon become dilated, and the ovum becoming uncovered and without proper support, its early discharge would be insured. The process would probably be aided by reflex irritation from the neck, inducing contraction in the muscular walls of the body of the uterus.

I have met with at least one case of this kind in my own practice. Profuse flowing occurred toward the end of the third month of pregnancy, with the usual symptoms of expulsive effort. The placental attachment was distinctly felt just within the partly dilated os. Its artificial removal was followed by persistent and finally alarming hæmorrhage, which could not be controlled by ice in the vagina nor by the administration of ergot. An injection of dilute ferric persulphate at once and permanently arrested it.

Barnes, in defining the limits for the situation of the placenta in prævial presentations, does not require that it should overlap the os internum. If any portion of the placenta find attachment within his cervical zone, "whether it consist in flap encroachment downwards, or whether it be the entire placenta," the condition exists for all the phenomena of prævial presentation. His language on this point is obscure, but he leads us to infer that while the ovum is primarily situated above the internal os, the cavity of the cervix is invaded by the placenta in the process of development.

If we are to accept the diagram introduced in his work as an illustration of his views, the dangerous area of attachment, consists almost entirely of the cervical cavity. The fact of its situation *over* the neck is of no importance, in his estimation of the danger of hæmorrhage, provided there is no encroachment upon this cervical area.

Dr. Ch. Bell, in a paper read recently before the Edinburgh Obstetrical Society, a report of which I have seen in the *Obstetrical Journal* since the last meeting of this society, while refuting the opinion of Barnes in regard to a cervico-orificial placenta, it seems to me, falls into a like error in giving a definition of his own. His statement is that it is attached to the lower segment of the uterus immediately "within the internal os," which it may cover either partially or entirely, and its development goes on as when placed elsewhere *paripassu* with that of the uterus.

My own definition of placenta prævia would be implantation *over* the os internum within the cavity of the body of the uterus. It may have all degrees of extent in situation; the expressions "partial" or "complete" having practical value only in the unavoidable hæmorrhage which occurs at the time of labor.

### Cause of Hæmorrhage during Utero-Gestation.

The current opinion has been and is still held by some, that during the latter months of pregnancy the neck of the uterus undergoes rapid development. The area of the placental site in consequence becomes enlarged, and is finally torn asunder from the placental attachment, and hæmorrhage results. Even admitting an intra-cervical implantation, this explanation is not verified in the history of many cases which have come under observation.

If this be a law of uterine development, the physiological expansion of the neck, upon this hypothesis, must invariably be attended by hæmorrhage during the progress of pregnancy. But this is by no means the case. Clinical experience teaches that while ante-partum hæmorrhage frequently occurs in placenta prævia, it is not only uncertain as to time and frequency, but is occasionally absent. This fact alone, I may remark, is proof beyond question against the doctrine of development of the neck during the latter months of utero-gestation, concerning which there has been so much discussion.

Barnes, in treating of this subject, more with a desire, it seems to me, for originality than consistency, insists that the very reverse of these conditions occurs during the growth of the placenta. According to his view, the placenta is endowed with much greater vitality than the uterus. The area of uterine attachment does not keep pace with its own development. As a consequence, the placenta outgrows its base, springs away from its attachment, and hæmorrhage is the result.

Is not either of these hypotheses at variance with what seems to be an ordinary law of growth in the animal economy? A neoplasm or growth of any kind, physiological or pathological, although differing in structure from its base, maintains its equable relations with it in consequence of the blood supply upon which its vitality and increase of tissue depend. If the vascularity of the underlying tissue be lessened, from either natural or artificial causes, in like proportion will the part supplied by the ultimate distribution of the vessels be affected, and the reverse of these conditions also is true. In cases of incomplete abortion, where the placenta remains adherent after the embryo is destroyed, its nutrition is modified in proportion to the arrested development of the uterus. The structure is more compact, less vascular and the area of attachment is not increased.

A like example occurs in extra-uterine fœtation, where the placenta is implanted on tissue still less adapted to its growth than the cervical portion of the uterus. Strangely enough, Dr. Barnes cites instances of tubal pregnancy as a proof of the correctness of his opinion. "Hence," says he, "in the case of a tubal gestation, there comes a time when the growth of the ovum is so rapid that the tubal sac is unable to keep pace with it, and it bursts." I reply that it is not the placenta which bursts away from its basis of tubal tissue, and which is the point at issue. The accident is the result of increasing weight, rather than over-distension, the muscular fibers being unable to afford adequate resistance. In my opinion, therefore, these theories of unequal development of either placenta or uterus are physiologically untenable.

I hold to the doctrine entertained, perhaps, by a minority of obstetric physiologists, that the causes of hæmorrhage in prævial placenta, instead of being unavoidable, are strictly *accidental*, until near the end of gestation, when the painless tonic contractions set in, which precede the phenomena of active labor. One of these accidental causes is certainly the occasional premature dilatation of the supra-vaginal portion of the neck, rupturing some of the utero-placental vessels near the margin of the internal os.

This dilatation is entirely passive on the part of the neck; not caused from development of its tissues, but by becoming softened in the vital act of preparation for the passage of the fœtus, the weight from above presses it asunder and separates its attachments. Further, the situation of the placenta on the lower segment of the uterus, where the walls are subject to varying pressure against the brim of the pelvis, to which is added the weight of the fœtus above it, would furnish conditions especially favorable to accidental detachment. It is a well known fact, however, as I have already stated, that not infrequently the period of utero-gestation is completed in placental presentations without the occurrence of hæmorrhage.

### Source of Hæmorrhage.

Does the flow of blood come from the open uterine sinuses, or from the detached portion of the placenta? It can scarcely be said that the anatomy of the placenta is as yet perfectly understood. The doctrine, however, of a free maternal circulation in its substance is generally accepted. Utero-placental arteries, slender and tortuous and without contractile sheaths, carry arterial blood into the lacunæ, or blood-pools, which bathe the tufts of the chorionic villi. These pools empty into the utero-placental sinuses, or emulgent veins. Hence there is no capillary circulation in the maternal placenta, but a direct flow of arterial blood into sinuses of large caliber. These vessels of the placenta, even the arterial, if we are to accept the authority of Virchow, lose their coats entirely on leaving the surface of the uterus, and are mere expansions of the serotina in its sheath-like ramifications about the tufts of the chorion. While it is generally admitted by those who advocate a placental source, that a moderate hæmorrhage takes place from the open uterine sinuses, it is asserted that the dangerous loss occurs from the partially separated placenta. Simpson and Mathews Duncan are especial advocates of this doctrine of the placental source of the hæmorrhage.

I can only remark that, with my limited experience in this class of cases, I have come to no definite opinion on the subject, but have a bias in favor of the older doctrine of hæmorrhage direct from the uterus. This view is the most rational one, and the opposite is based on theory rather than proof. The main argument brought forward by its advocates is the fact that a backward flow must take place in the uterine sinuses; whereas, in the placenta, the flow is direct through channels of large caliber, aided by the *vis-a-tergo* of the maternal arteries.

We know, however, from experience, that the open uterine sinuses *are* the source of profuse hæmorrhage in atonic states of that organ after labor. Again, if the hæmorrhage occur by way of the utero-placental arteries, ought not the blood to be arterial instead of venous, as is commonly observed? I regard the question as yet an unsettled one, to be decided by further investigation.

### TREATMENT.

It is but seldom that a case of placenta prævia can be left entirely to nature. Active measures are generally required to avert the dangers of hæmorrhage, and the safety of the patient will be in proportion to the efficiency of the means used to prevent it. When labor sets in, the hæmorrhage must be regarded as unavoidable; because partial or complete separation of the placenta is inevitable. The expansion of the neck is not the only factor in causing it. The advancing part of the fœtus crowds the placenta downward, and assists in forcibly severing it from its attachments. Finally, either the entire placenta is detached and carried onward, or, if the situation has been partial, a flap is thrown down and the cervix freed from obstruction.

This is the spontaneous detachment which nature seeks to accomplish, and which no art can prevent. The physiological limit of the hæmorrhage must be regarded as dependent on the completion of this act, provided the uterus continues in a state of active contraction, and forces the head or breech onward over the open uterine sinuses. May we not here draw a practical inference for treatment? Is not the removal of the placenta to the extent of clearing the canal for the advancing fœtus, plainly indicated, in conjunction with other measures for securing a safe delivery?

Simpson, as a result of bed-side experience, decided to remove it wholly from the uterine cavity. The practical effect was, as he found, the arrest of the hæmorrhage. But, by this method, the child is inevitably lost, and on this account, if on no other, the operation is objectionable, if other measures can save it, especially as in some cases, malposition of child or other conditions may compel a resort to version when, if living, a safe delivery might be possible. Barnes, on the other hand, contends that the entire detachment is not necessary for the arrest of the hæmorrhage, and he proposes the separation of the placenta over an area of nearly three inches from the os, and around the entire circumference. This treatment is based on the belief that there is an anatomical limit to the area of hæmorrhage, and when this part is freed from placental attachment, reaction occurs to such an extent that the uterine sinuses are closed. This, however, is mere hypothesis, and I think further experience will demonstrate that it is founded on erroneous views, and that such a procedure oftener increases than lessens the hæmorrhage. Would it not be more judicious management, having in view the two-fold object of arresting hæmorrhage and clearing the cervical canal, to separate the placental attachment fully one-half the circumference of the neck, and when practicable, not only to rupture the membranes, but to bring down the detached portion and thus, as nearly as possible, convert it into the accidental variety? When this is done, then, as in the accidental forms of hæmorrhage, the advancing head or breach would act as a compress upon the placental vessels, as well as covering the open mouths of the uterine sinuses.

The detachment of the placenta, however, either total or in part, in most cases is but one of the steps in the series of operative measures. No routine treatment can be adopted as a general rule of practice. Until the dilatation of the os is accomplished, either by artificial or natural methods, the hæmorrhage should be controlled by the vaginal or cervical tampon. The vaginal tampon, even if firmly applied, is apt to become loosened from the relaxation of the tissues, and besides the pressure upon the column of blood above the tampon has a hydrodynamic effect in forcing it around or through the meshes of whatever material may be used for the purpose. Of late years I have discarded the colpeurynters in use, as I believe they are inferior to firm packing with plugs of oiled cotton batting.

Theoretically, artificial dilatation of the os would seem preferable as it serves the additional purpose of arresting the hæmorrhage, but in practice the difficulty of keeping the dilator in proper position by reason of the nearness of the presenting part of the fœtus, makes the attempt very generally a failure. In whatever manner, however, the plugging may be done, it usually insures temporary safety until dilatation is accomplished.

Early rupture of the membranes, in my opinion, should be resorted to only in cases of partial presentation or, at least, where the placental edge can be brought down and as nearly as possible be converted into the accidental form of hæmorrhage. Barnes directs that if hæmorrhage be present the membranes should be ruptured as the first step in the management, before the os has become dilated and, even before the announcement of labor.

I think this advice, aside from the difficulty of the operation, as a general rule of treatment, should not be followed. Clinical experience has shown that the escape of the liquor amnii, with the placenta remaining implanted over the neck, will not always arrest the flooding. In that event, the attempt at version would be difficult, and in some cases would be impracticable.

Some authorities, instead of resorting to rupture of the membranes, as recommended by Barnes, discountenance it altogether. Simpson speaks doubtingly on the subject, and advises it only in cases of excessive quantity of fluid, or when hæmorrhage cannot be controlled, with an os too rigid to admit of version or other operative procedures.

In my opinion it ought never be done as a preliminary operation. The plug should be employed until partial dilatation is accomplished. If the membranes appear within the circle of the os, they should then be ruptured as in cases of accidental hæmorrhage. If this does not have the effect to check the flowing, turning can be promptly resorted to before the uterine walls contract to such an extent as to conform themselves rigidly to the inequalities of the fœtal surface. If the implantation be central, the membranes should be reached, after partial dilatation, by separation of the placental attachment with probe or finger.

Puncture of the placental mass is of doubtful propriety. It cannot well be done without recourse to a pointed instrument, which might injure the child; and more especially the fœtal circulation would be opened, and its death made certain from hæmorrhage.

In the following brief report of a case it will be seen that the usual measures were employed, but failed to save the patient, although the peculiar dyscrasia existing at the time of labor, doubtless contributed greatly in bringing about a fatal result.

Mrs. H., aged forty, mother of four living children, became pregnant for the fifth time in the spring of 1874. During the earlier part of her married life, she enjoyed ordinary health, but in later years had been a great sufferer from dyspepsia, and was also subject to nervous disorders. The stomach at times would tolerate only the simplest kinds of food, and during the months of her last pregnancy she had subsisted chiefly on raw eggs, milk with lime water, and occasionally, stimulants.

For years she had been subject to profuse menstrual discharge, amounting at times to flooding. These attacks caused debility, but she usually rallied rapidly, and had come to regard them with indifference. A state of hydræmia seemed to be established, and the impoverished state of the blood and defective nutrition were apparent in laxity of tissue and in general emaciation of the body. This lack of tissual tonicity had been noticed especially during a previous confinement, at which time she first came under my care.

As the os dilated, thrombi formed in both uterine labia; the anterior, about two-thirds the size of an orange, descended so as to lie outside the vulva, at the moment of expulsion of the head. The placenta came away promptly, and while no great hæmorrhage followed its removal, the drainage persisted to a moderate degree, much beyond the ordinary puerperal term. The condition of the neck at the time gave me uneasiness, as I thought its tissues must have been greatly damaged by the extravasation; but the tumefaction disappeared, and as far as I could determine, with but little sloughing of the parts.

In her last pregnancy, the first indication of placenta prævia occurred about forty-eight hours previous to her confinement, when on rising in the morning, she was taken with painless and profuse flooding. Her husband came for me immediately, but being ill at the time, at my request, he went for Prof. Nelson, who on arrival found that the hæmorrhage had ceased. On examination, he found the os high up and undilated. Pushing his finger well into the neck, he detected the presence of placental tissue. He explained to the husband the probable condition, enjoined quiet, and directed that one of us should be notified promptly upon the slightest return of flooding, or if any symptoms of labor should occur. At the end of forty-eight hours he came to my house, and stated that pains had set in and there was flowing, but he thought not excessive. I hurried to the house, taking with me my obstetric bag, containing, besides the usual instruments, a colpeurynter, Barnes' dilators, and a Davidson syringe, to which a flexible catheter was attached for injecting the uterus. I found the patient flowing freely, and already complaining of faintness. Pains short, sharp and frequent. On touching, the os was found near the brim, rigid, and dilated to the extent of readily admitting the index finger. By partly pushing the hand in the vagina, placental tissue could be felt overlying the neck of the uterns. I sent the husband hurriedly for assistance, giving him the addresses of a couple of the nearest physicians. The patient herself was perfectly calm, although she fully understood the critical nature of her condition. She was a lady of more than ordinary intelligence, had some knowledge of medicine, and between the time of Dr. Nelson's visit and that morning had read the entire article on placenta prævia in Meigs' Obstetrics. With the assistance of a couple of ladies who were neighbors, the hips were elevated and the head lowered. My first attempt was the introduction of Barnes' smallest dilator, but it resulted in failure. It may have been lack of skill, but I was embarrassed by the high situation of the os, and the placenta seemed to be crowded so closely to its edge that I could not keep it supported in position sufficiently to be caught by distension. The rubber colpeurynter was then introduced, covered with a linen handkerchief, and pushed well up into the vagina. Attaching the syringe it was filled with water to as great an extent as I dared without danger of its bursting. A dose of ergot with brandy was given, and a towel wrung out in cold water applied over the hypogastrium.

Under the action of the ergot, the pains increased and blood began to flow out of the vagina in spite of pressure and additional plugging. I now decided to resort to Barnes' method of separation of the placenta, and if possible to rupture the membranes. Hastily clearing the vagina, I introduced my hand well into its cavity, and finding the os a little more dilated, I easily swept the index finger its entire length, as I thought, around the circumference and separated the placental attachments. I could not reach the membranes, and the presenting part embarrassed the attempt as it pressed very strongly downward. I waited a few minutes, and found that the hæmorrhage instead of lessening had increased. The flow was constant, and it was noticed especially that the increase occurred in the interval immediately after the uterine contraction had ceased. Again the tampon was introduced as firmly as before, packing the vagina, however, with plugs of cotton batting squeezed out in soapy water. I intended that this should control the hæmorrhage for a time, until the os became further dilated; and, besides, the return of the husband with some one for assistance and counsel was momentarily expected.

I was confident that the head was downward, for the feet could be felt near the fundus, and movements indicated that the child was living. The pains at this stage were frequent, and she made attempts occasionally at bearing down which, though feeble, gave me hope that the os was dilating. The oozing from the vulva was moderate, and had it not been for the already enfeebled condition of the patient, it would not have especially added to the danger. But, slight as it was, and notwithstanding stimulation, she showed signs of increasing exhaustion. In addition to faintness she complained of oppression in breathing, and the uterus began to act more feebly. To add to my embarrassment the husband returned from a fruitless search for physicians. It seemed as if there was not a moment to be lost. Giving a dose of ergot with brandy and coffee, and allowing a very slight inhalation of ether, for a second time I cleared the vagina, and, with but little delay, dilated enough to admit my hand into the uterus, and passing toward the right groin, reached the membranes and ruptured them, the head being left occipito-anterior. Notwithstanding my arm nearly filled the os, the flow of blood at this moment was frightful. The fingers were hooked over the knees and version very easily accomplished. As the hand was withdrawn, the placenta in some manner became detatched and came with it. The thighs having been brought through the os, I waited and kept up pressure over the uterus, feeling secure for the time from hæmorrhage, and regarding a little delay as important to avoid the additional shock of sudden delivery. The pains were slight and without expulsive effort on the part of the patient. The shoulders were finally brought out but the head being large, hung firmly in the lower strait, and getting no assistance from the patient, I quickly delivered it with the forceps. The child was well developed and weighed, as estimated, about eleven pounds. The uterus could now be felt moderately well contracted, but the oozing continued like a reddish watery discharge. To add to the list of my misfortunes in the case, I found that in my haste, I had seized a bottle of tincture of iodine instead of the liquid persulphate of iron. I had, however, but recently noticed in the Transactions of the New York Obstetrical Society, published in the *Journal of Obstetrics*, that iodine was recommended as a substitute for the iron on account of its antiseptic properties, and that it was equally effective as a styptic. This I diluted in the proportion of about six to one with water, and injected into the uterine cavity. The flowing ceased but the patient was pulseless, indeed, as I thought, *in articulo mortis*. Brandy and coffee were injected into the rectum, as the stomach rejected the stimulants previously given. The patient had retained her consciousness perfectly, and had scarcely uttered a complaint during the entire struggle. At this moment, to a remark made to her husband, that I feared she was dying, she whispered amid her gaspings: "I hear, what you say, no, no, I have been like this before." Pressure was kept up over the abdomen, and heat applied as much as her restlessness would permit. At the end of half an hour the pulse was felt with more distinctness, and the extremities became warmer. Exhausted as I was, I almost sank in dismay as again I saw a stream of blood flowing out of the vagina, although the outline of the uterus was well defined, and seemed to be in a state of contraction. The diluted iodine was again injected, but death ensued within an hour after delivery.

This single-handed effort to save from death, but ending in failure, I have regarded as the most terrible experience of my professional life. In the light of that experience I should have done somewhat differently could the experiment have been repeated; but to some extent it exemplifies a fact which, I think, we occasionally realize in practice, that while the skill of the physician is often at fault, the resources of his art will sometimes fail him as well.

